# [Expression of Concern] PAT protein mRNA expression in primary rat hepatocytes: Effects of exposure to fatty acids

**DOI:** 10.3892/ijmm.2026.5995

**Published:** 2026-09-21

**Authors:** Elena Grasselli, Adriana Voci, Carlo Pesce, Laura Canesi, Emilia Fugassa, Gabriella Gallo, Laura Vergani

Int J Mol Med 25: 505-512, 2010; DOI: 10.3892/ijmm_00000370

Following the publication of the above paper, it was drawn to the Editor's attention by a concerned reader that, regarding the ORO-stained hepatocyte images shown in Fig. 3B on p. 510, the 'C 12h' and 'C 36h' data panels were strikingly similar in appearance (albeit one panel was sized slightly differently relative to the other), suggesting that the data in this figure had been assembled incorrectly.

The authors have been contacted by the Editorial Office to offer an explanation for the apparent data duplication in Fig. 3B in this paper, and we are awaiting their response. Due to the fact that we have been made aware of potential issues surrounding the scientific integrity of this paper, we are issuing an Expression of Concern to notify readers of this potential problem while the Editorial Office continues to investigate this matter further.

